# The non-anticoagulation costs of atrial fibrillation management: findings from an observational study in NHS Primary Care

**DOI:** 10.7573/dic.212254

**Published:** 2014-04-09

**Authors:** George Kassianos, Chris Arden, Simon Hogan, Laura Baldock, Ahmet Fuat

**Affiliations:** 1 The Ringmead Medical Practice, Bracknell, UK;; 2 Park Surgery, Chandlers Ford, UK;; 3 Sanofi, Guildford, UK;; 4 pH Associates Ltd, Marlow, UK;; 5 Carmel Medical Practice, Darlington, UK

**Keywords:** atrial fibrillation, resource use, primary health care, health care costs, observational study

## Abstract

**Background:**

Atrial fibrillation (AF) management represents a significant burden on the UK NHS. Understanding this burden will be important in informing future health care planning and policy development.

**Aim:**

To describe the non-anticoagulation costs associated with AF management in routine UK clinical practice.

**Materials, patients and methods:**

A retrospective observational study of 825 patients with AF undertaken in eight UK primary care practices. Data collected from routine clinical and prescribing records of all eligible, consenting patients, for a period of up to 3 years. The first 12 weeks following diagnosis was defined as the ‘initiation phase’; the period after week 12 was defined as the ‘maintenance phase’.

**Results:**

Mean (SD) total cost of AF management was £941 (£1094) per patient in the initiation phase and £426 (£597) per patient-year in the maintenance phase. AF-related inpatient admissions contributed most to total costs; the mean (SD) total cost per patient in the initiation phase was £2285 (£900) for admitted and £278 (£252) for non-admitted patients. Mean maintenance phase costs per year were £1323 (£755) and £168 (£234), respectively, for admitted and non-admitted patients. Significant patient variables contributing to high cost in the initiation phase were hypertension and younger patient age, although only accounting for 6% of cost variability. Significant variables in the maintenance phase (18% of cost variability) were the presence of congestive heart failure, structural heart disease or diabetes and the frequency of day case admissions, ECGs and hospitalisations in the initiation phase.

**Conclusions:**

Inpatient admissions contributed most to total AF management costs. Given the burden of hospital care, future work should focus on evaluating the appropriateness and reasons for hospitalisation in patients with AF and the factors affecting length of stay, with the aim of identifying opportunities to safely reduce inpatient costs.

A number of significant patient characteristics and initiation phase variables were identified, which accounted for 18% of the variability in total maintenance phase costs. However, none of these could adequately predict high maintenance phase costs.

## Introduction

The management and treatment of atrial fibrillation (AF) represents a significant resource use burden on the UK National Health Service (NHS), both in primary and secondary care. The Office of Health Economics estimates that in 2008 the total direct cost of AF to the NHS was £429 million; there were approximately 851,095 GP visits attributable to AF and patients with primary or secondary diagnoses of AF occupied an estimated 5.7 million bed-days [1]. The cost per AF-related stroke is estimated at £11,900 in the first year alone following stroke occurrence [2]. With the incidence and prevalence of AF predicted to rise significantly in the coming years due to an ageing population [3] and improving survival from underlying conditions closely associated with AF [4,5,6], this economic burden has the potential to increase significantly unless management efficiencies are made.

Given the economic burden of AF on the NHS, it is important that GPs and commissioners focus on measures to reduce AF-related health care costs while maintaining or improving quality of care, through service improvements and more effective diagnosis, treatment and management of patients with the condition, and effective anticoagulation. This will become increasingly important in the coming years, with the NHS under pressure to make significant efficiency savings and the UK government proposing major reforms which will change the way in which health care services are commissioned, organised and delivered.

A clear understanding of current AF management pathways and resource use will be important in informing future health care planning and policy development. Whilst existing data provide an estimate of the total cost of AF to the UK health care system, there are currently only limited international data [7–11] and no published UK studies describing the per-patient costs, differences between practices and the variables contributing to high resource use in ‘real world’ clinical practice.

The aim of this observational research study was to describe the non-anticoagulation NHS costs associated with the management of AF in routine UK clinical practice. No attempt has been made to describe the costs of anticoagulation for AF. There are many different models of anticoagulation care, including traditional secondary care models and primary care (GP)-based services [12,13], and there is evidence of variation in the quality and effectiveness of these services throughout the country [12]. Anticoagulation therapy is a rapidly changing area of practice, with the advent of new oral anticoagulants and an expected increase in the uptake of anticoagulation. There have also been developments such as patient self-testing [14] and the National Institute for Health and Care Excellence (NICE) have recently issued commissioning guidance to support the redesign of local care pathways [12], taking account of NICE recommendations for the novel oral anticoagulants. With many changes expected in this area of practice in the forthcoming years, any description of anticoagulation costs based on current practice is likely to be quickly superseded.

## Materials and methods

### Study design and methodology

This observational study was conducted by retrospective review of the clinical records of patients diagnosed with AF in eight general medical practices in England (n=7) and Wales (n=1), selected to ensure a good geographical spread and a mixture of urban and rural practices. Brompton, Harefield and NHLI Research Ethics Committee (REC) and local management approval were given to conduct the study in the participating primary care trusts.

The general practitioner (GP) investigator at each site identified candidate patients by searching the electronic patient records for currently registered patients aged ≥18 years at diagnosis, with a diagnosis of AF by Read codes for AF, AF with flutter, persistent AF, permanent AF and paroxysmal AF. Patient consent was sought via post by the GP for access to their records by a researcher, who then checked eligibility in the consenting patients against the remaining inclusion and exclusion criteria. Patients with secondary AF, those diagnosed <12 weeks before data collection or with no diagnosis date were excluded. A sample size of 1000 patients (500 initiation 500 maintenance) was chosen to ensure confidence intervals of ±5% on estimates of proportions, also allowing for a cluster effect of 1.2 due to the multicentre design.

Anonymised coded data were collected retrospectively from the primary care clinical records to describe resource use including AF-related prescribing, primary and secondary care visits and hospitalisations and demographics. Data were collected between March and August 2010. For each patient, data were collected for the most recent 3 years of management (i.e. 2007–2010) or from the date of diagnosis of AF, if the patient was diagnosed <3 years before data collection.

For the purposes of this study the first 12 weeks of management following diagnosis was referred to as the ‘initiation phase’. The period from week 12 onwards was referred to as the ‘maintenance phase’. This distinction was made because it was recognised that AF resource use was likely to be higher in the period immediately following diagnosis. For patients who had been recently diagnosed with AF (<9 months before data collection), data were collected on the initiation phase only. For patients diagnosed more than 3 years before data collection, data were collected on the most recent 3 years of management (i.e. maintenance phase only), whilst patients diagnosed 9 months–3 years before data collection provided data on both the initiation and maintenance phases of management.

### Analysis methodology

Data were analysed using Microsoft Excel^®^ and SPSS^®^ according to a pre-agreed analysis plan. The main endpoint was NHS resource use, broken down by initiation and maintenance phases of management.

Resource use in the initiation phase is expressed as resource use per patient within a 12-week period. Resource use in the maintenance phase is expressed as resource use per patient-year, with data adjusted for patients providing between 6 and 12 months of maintenance data.

Costs were assigned to AF-related primary care visits, secondary care attendances, hospitalisations, investigations and blood tests, using published NHS reference costs [15–19] (Table 1). Where more than one reference cost was available for a particular aspect of management, the mean cost was used. A single cost (i.e. the national average unit cost for a non-elective inpatient admission for arrhythmia or conduction disorder) was assigned to each AF-related hospitalisation, irrespective of the length of stay or any procedures/investigations performed during the admission. The costs for AF medications were assigned using the prices published in BNF 61, March 2011 [20] (Table 2). Drug doses were not recorded for the study and therefore costs were assigned for each medication using a presumed daily dose (the midpoint of all the recommended doses published in BNF 61 for that medication). Route of administration was also not recorded for the study and therefore costs were assigned for oral tablets/capsules, using the average of the minimum and maximum costs of all the generic and proprietary versions of each drug (Table 2).

The costs of AF-related primary and secondary care attendances, hospitalisations, investigations, blood tests and AF medications, were used to calculate a total cost per patient for the initiation and maintenance phases of management as applicable. All anticoagulation costs (i.e. visits for anticoagulation, INR tests and antiplatelet/anticoagulant medication) were excluded from the analysis.

Analysis of AF costs included stratification by type of AF, by centre, and by patients who did and did not require inpatient hospitalisation. Costs by AF type were analysed using Analysis of Variance (ANOVA).

Costing data were analysed using multiple regression to ascertain which combination of variables contributed most to the total costs during both the initiation and maintenance phases of management. Analysis of initiation phase costs was carried out for all patient-related independent variables; analysis of maintenance phase costs included both patient-related and initiation phase variables. Stepwise regression was carried out whereby at each stage the least significant variable of the previous stage was removed before the next variable that most contributed to the explanation of the variability of the dependent variable was added. This continued until no other variable contributed to the explanation with a significance of *p*<0.05.

## Results

### Participating practices

Eight general medical practices of 5–10 GPs with a median list size of 13,440 (range 6500–15,500) participated, with three practices including a GP with a special interest (GPwSI) in the management of AF.

There were 1245 patients across all the participating practices who met the eligibility criteria and all were approached for consent. Data were collected from all 825 (66%) consenting patients. The number of patients per practice ranged from 82 to 151. Data were available on the initiation phase from 310 patients and the maintenance phase from 769 patients. Two hundred and fifty-four patients provided data on both phases.

### Patient characteristics

There were 462 (56%) male patients. At the time of diagnosis 17% of patients (137/819 with data available) were aged less than 60 years and 682 (83%) were aged 60 years or over. The mean age at diagnosis was 70.5 years (SD 11.1; range 22.4–95.7).

At the time of data collection 363 (44.0%) patients had a current diagnosis of permanent AF (i.e. an ongoing long-term episode), 299 (36.2%) paroxysmal AF (i.e. recurrent episodes that self-terminate in <7 days), 55 (6.7%) persistent AF (i.e. recurrent episodes lasting >7 days) and 46 (5.6%) patients first-detected AF (i.e. only one diagnosed episode). Fourteen (1.7%) patients had a record of two types of AF and 48 (5.8%) patients had no record of AF type.

### AF-related resource use

Details of the numbers of visits to primary and secondary care, and inpatient hospital admissions are summarised in Table 3. Table 4 shows the number of inpatient hospitalisations by reason.

Table 5 shows the AF-related blood samples, investigations and procedures performed during both the initiation and maintenance phases of management. This includes the number and percentage of patients undergoing each test and the mean (SD) number performed per patient.

### Cost of AF management

**Figure 1.**
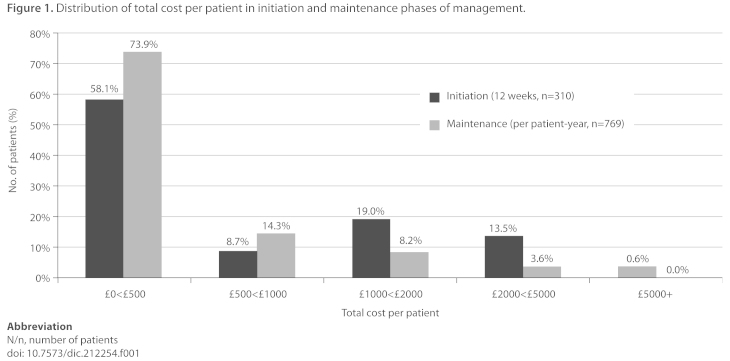
Distribution of total cost per patient in initiation and maintenance phases of management. Abbreviation N/n, number of patients doi: 10.7573/dic.212254.f001

The estimated total cost of AF management per patient in both the initiation and maintenance phases, and the estimated cost for each separate aspect of AF management, is presented in Table 6 and Figure 1.

The mean total cost of AF management was £941 per patient in the initiation phase (SD £1094, range £0–£7285) and £426 per patient per year in the maintenance phase (SD £597, range £0–£4961). The cost of inpatient admissions contributed most to total management costs; the mean (SD) total cost of AF management per patient in the initiation phase was £2285 (£900) for patients with inpatient hospitalisations during this period and £278 (£252) for patients with no inpatient hospitalisations. The mean (SD) maintenance phase costs for admitted and non- admitted patients, respectively, were £1323 (£755) and £168 (£234) (Table 7).

### Variables contributing to cost variability – multivariate analysis

Table 8 shows the variables contributing most to total cost during both the initiation (Table 8 part a) and maintenance (Table 8 part b) phases of management. The regression coefficients show the added initiation phase cost (£) associated with each variable.

In the initiation phase, the variables contributing most to total cost were age at diagnosis (total initiation phase costs reduced by £16 with each increasing year of age) and hypertension (adding £458 to total costs) (Table 8 part a). However, these variables combined only explained 5.6% of the variability in initiation phase costs (r^2^ coefficient of multiple determination=0.056) with the remaining 94.4% unknown (i.e. due to either random variation or factors which were not assessed in this study).

In the maintenance phase, the patient variables contributing most to total cost were the presence of congestive heart failure (+£148), structural heart disease (+£155) and diabetes (+£180) (Table 8 part b). The presence of dyslipidaemia reduced maintenance phase costs by £95 per year. The numbers of hospitalisations, ECGs and day case admissions in the initiation phase were also associated with higher maintenance phase costs, with each hospitalisation adding £360, each ECG adding £95, and each day case admission adding £807 to the total cost for this period. All of these patient and initiation phase variables combined explained 18% of the variability in maintenance phase costs (r^2^ coefficient of multiple determination=0.183), with the remaining 82% unknown (i.e. due to either random variation or factors which were not assessed in this study).

### Cost of AF management by AF type

The mean (SD) cost per patient in the initiation phase was £981 (£887) for first detected AF, £927 (£1201) for paroxysmal, £585 (£688) for persistent and £959 (£1014) for permanent AF. Although the mean initiation phase cost for patients with persistent AF was lower than those of the other three groups, overall there was no significant difference in costs by AF type in the initiation phase (*p*=0.53).

The mean (SD) cost per patient per year in the maintenance phase was £108 (£348) for first detected, £407 (£584) for paroxysmal, £467 (£660) for persistent and £339 (£550) for permanent AF. Overall, there was a significant difference in maintenance phase costs per year by AF type (*p*=0.005). The mean maintenance phase cost for patients with first detected AF was significantly lower than the cost for patients with all other AF types (*p*<0.01), with no significant difference in cost between the subgroups of patients with paroxysmal, persistent and permanent AF (Table 9).

### Cost of AF management by centre

**Figure 2.**
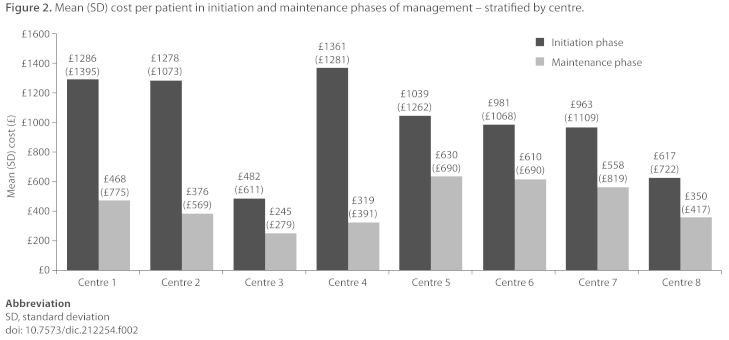
Mean (SD) cost per patient in initiation and maintenance phases of management – stratified by centre. Abbreviation SD, standard deviation doi: 10.7573/dic.212254.f002

The mean (SD) total cost of AF management per patient in the 12-week initiation phase ranged between £482 (£611) (Centre 3) and £1361 (£1281) (Centre 4). The mean (SD) cost per patient per year in the maintenance phase ranged from £245 (£279) (Centre 3) to £630 (£690) (Centre 5) (Figure 2). Table 10 shows the mean (SD) cost of each component of AF management, stratified by centre.

## Discussion

This paper reports an analysis of the non-anticoagulation costs associated with the management of AF, from the results of a retrospective observational research study of 825 patients with AF, recruited from eight primary care practices in England and Wales.

As expected, inpatient admissions and secondary care attendances were the highest-costing components of AF management; the mean total cost per patient in the initiation phase was £2285 for patients with one or more inpatient hospitalisations during this period and just £278 for those with no inpatient hospitalisations. Findings for the maintenance phase were similar, with a mean cost per patient-year of £1323 and £168, respectively, for hospitalised and non-hospitalised patients. This finding is in line with results of previous studies conducted in the USA [10] and other European countries [7–9,11], which also found hospitalisations to contribute most to overall costs. With one-third of patients in the current study (33%) admitted to hospital during the initiation phase and 18% during the maintenance phase, mean lengths of stay of 5.6 and 6.4 days (initiation and maintenance phase, respectively), and recently published work suggesting that AF-related hospitalisations are increasing at a faster rate than those for other cardiovascular diseases [21], hospital care for patients with AF clearly represents a considerable burden of health care resource and is an area which may be targeted to reduce overall costs. However, to ensure that cost-cutting measures do not compromise quality of care, further research is needed to evaluate the appropriateness and reasons for hospital admissions in patients with AF and the factors affecting length of stay; often it may be the patient’s other comorbidities or social care needs which are the determining and limiting factor.

The between-patient range of costs was high, with the care of most patients (58% in the initiation phase and 74% in the maintenance phase) costing little (<£500 per patient/per patient-year) and a only a small number of patients (14% in the initiation phase and 4% in the maintenance phase) at the high end of the distribution (i.e. care costing more than £2000 per patient/per patient-year). Whilst inpatient admissions were the highest-costing individual component of AF management, most patients had no inpatient admissions in either phase of management.

The presence of a small number of patients at the high end of the cost distribution led to our attempt (using multiple regression analysis) to identify factors predictive of high cost. The patient and initiation phase variables which correlated most with maintenance phase costs (accounting for 18% of variability) were the presence of congestive heart failure, structural heart disease and diabetes, and the number of hospitalisations, ECGs and day case admissions in the initiation phase. The correlation between cardiac comorbidities and higher maintenance phase costs is consistent with the findings of studies conducted previously in other European countries [7,9]. However, it is very difficult to separate AF-related resource from that which is attributable to other comorbidities, and although the aim of this study was to record only AF-related resource, hospitalisations associated with other cardiac comorbidities (where AF was a secondary diagnosis or contributing factor) were sometimes also recorded. In these cases it is not known how much the diagnosis of AF contributed either to the initial hospital admission or length of stay. The correlation between initiation phase resource use (number of hospitalisations and day case admissions) is interesting to note, and suggests that patients who require secondary care intervention (particularly planned day case admissions) in the first 12 weeks following diagnosis will continue to have high costs in the period following this and may therefore warrant more intensive primary care management to try to avoid unplanned secondary care resource use. It should be noted, however, that only a small proportion of patients (4%) had day case admissions in the initiation phase. The patient variables contributing most to total costs in the initiation phase were younger patient age and the presence of hypertension. However, these variables combined explained only a very small proportion (6%) of the cost variability for this period. The correlation between younger patient age and increased initiation phase costs may indicate relatively more intensive management in younger patient groups, perhaps in terms of more frequent primary care review, earlier referral into secondary care or more intensive medication management. The finding cannot be explained by the higher use of interventions such as electrical cardioversion and cardiac ablation, as the use of these procedures was very low overall but particularly so in the initiation phase.

Analysis of AF management costs by AF type showed that although the mean initiation phase cost was lower for persistent AF (mean £585) than for paroxysmal (mean £927), permanent (mean £959) or first detected AF (mean £981), there was overall no significant difference between the groups (*p*=0.531). In the maintenance phase the mean cost per patient per year was significantly lower for patients with first detected AF (£108) than for patients in any other AF category (*p*<0.01). However, this is not surprising given that patients in this category have had (by definition) only one diagnosed AF episode. There were no significant differences in maintenance phase costs between the subgroups of patients with paroxysmal, persistent and permanent AF, suggesting that once an appropriate treatment strategy is determined and patients are established on medication, the management costs for the different types of AF are comparable.

There was wide variation in initiation and maintenance phase costs between centres, with mean initiation phase costs per patient ranging from £482 (Centre 3) to £1361 (Centre 4) and mean maintenance phase costs per patient per year ranging from £245 (Centre 3) to £630 (Centre 5).

## Strengths and limitations

The primary strength of this study is that it provides a description of the costs of AF management in ‘real world’ UK clinical practice; however, there are a number of limitations. As data were collected retrospectively from primary care records, the data quality relied heavily on the level of detail routinely recorded in these records and on the information provided in correspondence from secondary care.

The number of blood tests and procedures recorded (in particular ECGs during the initiation phase) is likely to be underestimated, since some patients will have been diagnosed and had an ECG whilst in hospital (details of which may not have been recorded in the primary care records). In addition, a number of patients may have had ECGs carried out prior to diagnosis.

This study may provide an overestimate of the cost of hospital care in patients with AF. Data collectors were instructed to record ‘AF-related’ inpatient admissions; however, it is very difficult to separate AF-related resource from that which is attributable to other comorbidities, and for some recorded hospitalisations AF may have been a secondary diagnosis.

There are also limitations with the costing data. Drug costs were assigned using the BNF reference costs and it is acknowledged that these may not reflect the NHS purchase price. Data on drug doses and route of administration were not captured during the study and therefore the cost assigned to each medication was based on the cost of oral tablets/capsules for an assumed average daily dose. The average of the minimum and maximum costs of all generic and proprietary versions of each drug was used in calculations.

Inpatient stays were costed per episode, using a single cost assigned to an AF-related inpatient stay, and therefore the varying lengths of hospital stay and any operations, procedures or investigations performed during the admission, were not accounted for.

## Conclusions

This study confirms that inpatient admissions and secondary care attendances are the largest contributors to total AF management costs, both in the first 12 weeks after diagnosis and in the period following this. Whilst this finding is not unexpected, it is helpful to quantify, and will be useful to commissioners and health care providers in discussions about how AF management can be made as efficient as possible. Given the burden of hospital care, health care professionals should review the appropriateness and reasons for hospital admissions in patients with AF and the factors affecting length of stay. Future work should focus on identifying opportunities to safely reduce avoidable hospital admissions and length of hospital stay, ensuring that patients are still referred to secondary care when appropriate. Further research is also warranted to explore the link between cost and patient outcomes, as this was beyond the scope of the current study.

The study identified a number of significant patient characteristics and initiation phase variables, which accounted for 18% of the variability in total maintenance phase costs. However, they could not predict high resource users in the maintenance phase and hence identify those for whom greater primary care intervention may reduce hospitalisations.

## Figures and Tables

**Table 1.** Costs assigned to each component of AF management [15–19].

**Component of AF management**	**Cost assigned (£)**
**Primary and secondary care attendances**	
Primary care visit	£32.00
Outpatient visit	£152.00
Day case admission^*^	£637.00
Inpatient admission	£1534.00
A&E attendance	£97.00
**Investigations/procedures**	
Electrocardiogram (ECG)	£32.50
Holter ECG	£59.79
Chest x-ray	£86.52
Echocardiogram (ECHO)	£76.00
**Blood tests**	
Electrolytes	£4.11
Full blood count (FBC)	£4.57
Renal function	£4.11
Thyroid function test	£3.68

* Patient admitted electively for care which does not require a hospital bed overnight.

**Abbreviation**

AF, atrial fibrillation

doi: 10.7573/dic.212254.t001

**Table 2.** Costs assigned to AF medications [20].

**Drug name**	**Presumed average daily dose**	Average daily cost^*^
Amiodarone	200 mg	£0.16
Atenolol	75 mg	£0.09
Bisoprolol	10 mg	£0.25
Carvedilol	25 mg	£0.23
Co-tenidone	62.5 mg	£0.05
Digoxin	125 μg	£0.03
Diltiazem	240 mg	£0.38
Disopyramide	500 mg	£1.15
Flecainide	200 mg	£0.42
Labetalol	400 mg	£0.26
Lercanidipine	10 mg	£0.20
Metoprolol	150 mg	£0.13
Nebivolol	5 mg	£0.25
Nifedipine	30 mg	£0.21
Propafenone	450 mg	£0.24
Propranolol	80 mg	£0.03
Sotalol	240 mg	£0.22
Verapamil	240 mg	£0.07

* Average of the minimum and maximum costs of all generic and proprietary versions.

**Abbreviation**

AF, atrial fibrillation

doi: 10.7573/dic.212254.t002

**Table 3.** Health care visits for AF.

**Visit type**	**Initiation phase (n=310)**		**Maintenance phase (n=769)**	
	
	**N (%) of patients**	**Mean no. visits per patient (SD)**	**N (%) of patients**	**Mean no. visits per patient-year (SD)**
**AF-related primary care visits**				
Patients with any primary care visit	253 (82%)	2.4 (2.2)	613 (80%)	1.5 (1.8)
• GP visits	250 (81%)	2.3 (2.1)	581 (76%)	1.4 (1.7)
• Nurse visits	45 (15%)	0.2 (0.5)	173 (22%)	0.2 (0.3)
**AF-related secondary care visits (excluding inpatient admissions)**				
Patients with any secondary care visit (excl. admissions)	196 (63%)^*^	1.1 (1.2)	427 (56%)	0.8 (1.0)
• Outpatient clinic	176 (57%)	0.8 (0.9)	384 (50%)	0.6 (0.9)
• Day case unit^*^	12 (4%)	0.0 (0.2)	52 (7%)	0.0 (0.2)
• A&E visit	26 (8%)	0.1 (0.5)	82 (11%)	0.0 (0.2)
• Other secondary care visit	3 (<1%)	0.0 (0.1)	2 (<1%)	0.0 (0.0)
**AF-related inpatient admissions**				
Patients with any inpatient admission	101 (33%)	0.4 (0.7)	139 (18%)	0.1 (0.3)
Mean (SD) length of hospital stay (days)	5.6 (7.8)	–	6.4 (9.6)	–

* A day case admission is where a patient is admitted electively for care which does not require a hospital bed overnight. The majority of day case

**Abbreviation**

A&E, accident and emergency; AF, atrial fibrillation; GP, general practitioner; N/n, number of patients; SD, standard deviation

doi: 10.7573/dic.212254.t003

**Table 4.** Number of patients hospitalised by reason.^*1^

**Recorded reason for hospitalisation**	Initiation phase (n=101)^*2^		Maintenance phase (n=139)^*2^	
	
	**N**	**%**	**N**	**%**
AF	25	25	11	8
Chest pain	9	9	18	13
Pacemaker	6	6	17	12
Palpitations	9	9	10	7
Shortness of breath (SOB)	8	8	10	7
Cerebrovascular accident (CVA)	3	3	11	7
Cardiac ablation	2	2	11	7
Fast AF	5	5	7	5
Myocardial Infarction (MI)	4	4	2	1
Angiogram	3	3	2	1
Coronary artery bypass graft (CABG)	2	2	3	2
Collapse	1	1	4	3
Heart failure	2	2	3	2
Chest infection	4	4	0	0
Fall	0	0	4	3
Palpitations + SOB	3	3	1	1
Acute heart failure	1	1	2	1
AF + congestive cardiac failure	3	3	0	0
Angina	2	2	1	1
Angiography	1	1	2	1
Arrhythmia	1	1	2	1
Breathlessness	1	1	2	1
Chest pain + SOB	1	1	2	1
GI bleed	0	0	3	2
PR bleed	0	0	3	2

*1 Partial table showing reasons with 3 or more patients associated.

*2 n is equal to the number of patients with one or more inpatient admissions in either the initiation or maintenance phase of management.

**Abbreviations**

AF, atrial fibrillation; CABG, coronary artery bypass graft; CVA, cerebrovascular accident; GI, gastrointestinal; MI, myocardial infarction;

N/n, number of patients; PR, per rectum; SOB, shortness of breath

doi: 10.7573/dic.212254.t004

**Table 5.** AF-related blood tests and investigations.^*1^

	**Initiation phase (n=310)**		**Maintenance phase (n=769)**	
	
	**N (%) of patients**	**Mean no. per patient (SD)**	**N (%) of patients**	**Mean no. per patient-year (SD)**
**AF-related blood samples**				
Any sample	118 (38%)	0.6 (1.0)	396 (51%)	1.1 (2.1)
• Electrolytes	102 (33%)	0.4 (0.7)	337 (44%)	0.6 (0.9)
• FBC	79 (25%)	0.3 (0.6)	306 (40%)	0.4 (0.7)
• Renal function	79 (25%)	0.3 (0.6)	246 (32%)	0.4 (0.8)
• Thyroid function	58 (19%)	0.2 (0.4)	224 (29%)	0.3 (0.5)
• Other	59 (19%)	0.2 (0.5)	226 (29%)	0.2 (0.5)
**Primary care AF-related investigations/procedures**				
• ECG	136 (44%)^*2^	0.6 (0.8)	213 (28%)	0.2 (0.3)
• Holter ECG	11 (4%)	0.0 (0.2)	17 (2%)	0.0 (0.1)
• Other	10 (3%)	0.0 (0.2)	20 (3%)	0.0 (0.1)
**Secondary care AF-related investigations/procedures**				
• ECG	63 (20%)	0.2 (0.4)	124 (16%)	0.1 (0.3)
• Holter ECG	28 (9%)	0.1 (0.3)	92 (12%)	0.1 (0.2)
• ECHO	88 (28%)	0.3 (0.5)	167 (22%)	0.1 (0.2)
• Chest X-ray	20 (6%)	0.1 (0.2)	53 (7%)	0.0 (0.1)
• Electrical cardioversion	8 (3%)	0.0 (0.2)	44 (6%)	0.0 (0.2)
• Pharmacological cardioversion	0 (0%)	–	1 (<1%)	0.0 (0.0)
• Other	0 (0%)	–	0 (0%)	–

*1 The number of blood tests and procedures recorded (in particular ECGs during the initiation phase) is likely to be underestimated, since some patients will have been diagnosed and had an ECG whilst in hospital (details of which may not have been recorded in the primary care records). In addition, a number of patients may have had ECGs carried out prior to diagnosis.

*2 All percentages are calculated out of the total number of patients (i.e. 310 in the initiation phase and 769 in the maintenance phase), NOT the number of patients who were referred to secondary care.

**Abbreviations**

AF, atrial fibrillation; ECG, electrocardiogram; ECHO, echocardiogram; FBC, full blood count; N/n, number of patients; SD, standard deviation

doi: 10.7573/dic.212254.t005

**Table 6.** Cost of AF management.

**Component of AF management**	**Cost per patient during initiation phase (n=310)**			**Cost per patient-year during maintenance phase (n=769)**		
	
	**Mean**	**SD**	Range^*^	**Mean**	**SD**	**Range**
Investigations (ECG, Holter ECG, ECHO, CXR)	£59	£60	£0–£243	£23	£36	£0–£240
Blood testing	£5	£9	£0–£50	£7	£11	£0–£75
AF medications	£9	£10	£0–£47	£49	£49	£0–£288
AF-related primary care visits	£78	£69	£0–£416	£49	£58	£0–£365
AF-related secondary care visits (outpatient, day case, A&E)	£166	£201	£0–£1426	£128	£206	£0–£1526
AF-related inpatient admissions	£624	£1006	£0–£6136	£170	£448	£0–£4768
**Total cost**	**£941**	**£1094**	**£0–£7285**	**£426**	**£597**	**£0–£4961**

* Where the total cost for a patient in either the initiation or maintenance phase was zero, this may be due to patients being diagnosed and treatments initiated in secondary care (details of which may not have been recorded in the primary care records). In addition, a number of patients may have had investigations and primary or secondary care visits prior to diagnosis.

**Abbreviations**

A&E, accident and emergency; AF, atrial fibrillation; CXR, chest x-ray; ECG, electrocardiogram; ECHO, echocardiogram; N/n, number of patients;

SD, standard deviation

doi: 10.7573/dic.212254.t006

**Table 7.** Costs of AF management, stratified by patients with and without inpatient admissions.

	**Cost per patient during initiation phase**				**Cost per patient-year during maintenance phase**			
	
	**n**	**Mean**	**SD**	**Range**	**n**	**Mean**	**SD**	**Range**
**Patients without inpatient admissions**	209	£278	£252	£0–£1716	630	£168	£234	£0–£2016
**Patients with inpatient admission**	101	£2285	£900	£1534–£7256	139	£1323	£755	£511–£4887

**Abbreviations**

N/n, number of patients; SD, standard deviation

doi: 10.7573/dic.212254.t007

**Table 8.** Variables contributing to total cost during the initiation and maintenance phases of management.

	Regression coefficient (£)^*^	**95% Confidence interval (£) (±)**	**Standard error**	*p*
**a) Patient variables contributing to total cost in initiation phase**				
Age at diagnosis (per year)	−15.85	11.50	5.85	0.01
Presence of hypertension	457.57	244.81	124.81	<0.001
**b) Patient characteristics and initiation phase variables contributing to total cost (per year) in maintenance phase**				
Presence of congestive heart failure	148.20	104.87	53.42	<0.01
Presence of structural heart disease	154.67	108.70	55.37	<0.01
Presence of diabetes	179.95	112.44	57.27	<0.01
Presence of dyslipidaemia	−94.54	87.63	44.64	0.034
No. hospitalisations in initiation phase (per hospitalisation)	359.85	92.50	47.11	<0.0001
No. of ECGs in initiation phase (per ECG)	95.45	79.31	40.40	0.018
No. day case admissions in initiation phase (per day case admission)	807.33	287.87	146.64	<0.0001

* Regression coefficients show the added initiation/maintenance phase cost associated with each patient or initiation phase variable.

**Abbreviation**

ECG, electrocardiogram

doi: 10.7573/dic.212254.t008

**Table 9.** Cost of AF management, stratified by AF type.

**AF type**	**Cost per patient during initiation phase**				**Cost per patient-year during maintenance phase**			
	
	**n**	**Mean**	**SD**	**Range**	**n**	**Mean**	**SD**	**Range**
First detected	27	£981	£887	£0–£3317	38	£108	£348	£0–£2025
Paroxysmal	112	£927	£1201	£0–£7256	273	£407	£584	£0–£3705
Persistent	20	£585	£688	£0–£2237	53	£467	£660	£0–£2598
Permanent	125	£959	£1014	£0–£3721	348	£339	£550	£0–£4887
**ANOVA**	*p*=0.531				*p*=0.005			

**Abbreviations**

AF, atrial fibrillation; ANOVA, analysis of variance; N/n, number of patients; SD, standard deviation

doi: 10.7573/dic.212254.t009

**Table 10.** Cost of each component of AF management, stratified by centre.

**Component of AF management**	**Centre 1**	**Centre 2**	**Centre 3**	**Centre 4**	**Centre 5**	**Centre 6**	**Centre 7**	**Centre 8**
**Mean (SD) cost per patient during initiation phase**								
Investigations	£81 (£69)	£64 (£68)	£36 (£41)	£83 (£78)	£44 (£47)	£75 (£70)	£76 (£58)	£43 (£46)
Blood tests	£7 (£9)	£7 (£9)	£0 (£0)	£1 (£3)	£5 (£7)	£11 (£13)	£9 (£10)	£4 (£7)
AF medications	£12 (£10)	£11 (£11)	£5 (£8)	£6 (£6)	£10 (£10)	£7 (£9)	£11 (£11)	£12 (£11)
Primary care visits	£63 (£59)	£92 (£50)	£60 (£56)	£58 (£57)	£81 (£78)	£112 (£98)	£96 (£65)	£62 (£57)
Secondary care visits	£147 (£196)	£183 (£176)	£186 (£184)	£168 (£225)	£164 (£260)	£172 (£248)	£195 (£184)	£102 (£168)
Inpatient admissions	£976 (£1261)	£920 (£953)	£195 (£516)	£1046 (£1196)	£736 (£1169)	£604 (£1010)	£575 (£1031)	£393 (£679)
**Mean (SD) cost per patient-year during maintenance phase**								
Investigations	£22 (£37)	£22 (£31)	£9 (£17)	£21 (£28)	£33 (£49)	£44 (£50)	£20 (£36)	£22 (£32)
Blood tests	£6 (£7)	£5 (£6)	£0.09 (£0.75)	£0.24 (£1.16)	£12 (£9)	£27 (£17)	£6 (£9)	£3 (£5)
AF medications	£71 (£62)	£51 (£47)	£40 (£41)	£34 (£34)	£51 (£56)	£43 (£50)	£54 (£42)	£51 (£53)
Primary care visits	£19 (£27)	£46 (£53)	£45 (£41)	£22 (£28)	£107 (£75)	£79 (£76)	£46 (£58)	£40 (£43)
Secondary care visits	£115 (£196)	£101 (£176)	£124 (£184)	£149 (£225)	£211 (£260)	£163 (£248)	£79 (£184)	£102 (£168)
Inpatient admissions	£235 (£666)	£152 (£382)	£27 (£137)	£93 (£259)	£216 (£469)	£252 (£469)	£353 (£673)	£133 (£331)

**Abbreviations**

AF, atrial fibrillation; SD, standard deviation

doi: 10.7573/dic.212254.t010

## Abbreviations

A&E: accident and emergency
AF: atrial fibrillation
ANOVA: Analysis of Variance
BNF: British National Formulary
CABG: coronary artery bypass graft
CVA: cerebrovascular accident
CXR: chest x-ray
ECG: electrocardiogram
ECHO: echocardiogram
FBC: full blood count
GI: gastrointestinal
GP: general practitioner
GPwSI: general practitioner with special interest
MI: myocardial infarction
NHLI: National Heart and Lung Institute
NHS: National Health Service
PR: per rectum
REC: research ethics committee
SD: standard deviation
SOB: shortness of breath.
